# Improving the Energy Conversion Efficiency of a Laser-Driven Flyer by an In Situ-Fabricated Nano-absorption Layer

**DOI:** 10.1186/s11671-020-03346-5

**Published:** 2020-06-05

**Authors:** Liang Wang, Yichao Yan, Xiangbo Ji, Wanli Zhang, Hongchuan Jiang, Wenzhi Qin, Yao Wang, Duo Tang

**Affiliations:** 1 State Key Laboratory of Electronic Thin Films and Integrated Devices, University of Electronic Science and Technology of China, Chengdu, 610054 China; 2 Institute of Chemical Materials, China Academy of Engineering Physics, Mianyang, 621900 China

**Keywords:** Laser-driven flyer, Nanostructured absorption layer, Energy conversion, PDV

## Abstract

Three kinds of Al flyer plates with different nanostructured absorption layers were in situ prepared by a direct laser writing technology to improve the energy conversion efficiency in a laser-driven flyer assembly. Microstructures, light absorption, and flyer velocity in the acceleration chamber were investigated. The reflectance for the flyers at 1064-nm wavelength can be reduced from 81.3 to 9.8% by the nanostructured absorption layer. The terminal velocity of a 50-μm-thick Al flyer irradiated by a 60-mJ laser pulse is 831 m/s, while the velocity of the flyer with an in situ-fabricated nano-absorption layer reaches up to 1113 m/s at the same condition. Resultantly, the energy conversion efficiency of the flyer with a nanostructure absorption layer can reach as high as 1.99 times that of the Al flyer. Therefore, the nanostructured absorption layer in situ prepared on the surface of a flyer provides a new method to significantly improve the energy conversion efficiency of a laser-driven flyer.

## Introduction

Laser-driven flyer (LDF) used for detonating explosives offers a promising approach to well-controlled, short-pulse shock compression of condensed phase materials [1–4]. In a LDF setup, a thin metal foil supported by a transparent window substrate is often launched by a nanosecond pulsed laser, a layer of the metal foil named the ablation layer is ablated generating high-pressure plasmas instantly, and the plasmas then drive the remains of the metal foil to fly at a velocity of several kilometers per second as a flyer. Metal aluminum is ideal as the flyer material due to its good tenacity and low density. However, since a large fraction of energy is lost due to high reflection of the pure aluminum flyer, the energy conversion efficiency of the flyer (defined as the ratio between the flyer kinetic energy and the incident laser energy) is extremely low, which has greatly limited the practical applications of the LDF [5, 6].

Plenty of works have been carried out for the purpose to improve the energy conversion efficiency of LDF. Considering the energy conversion efficiency could be improved by introducing a layer with stronger absorption at the incident laser wavelength due to decreased reflection [7], many materials with lower reflectivity compared to pure aluminum have been studied as the absorption layer. Labaste et al. [8] and Brierley et al. [9] investigated several materials as absorption layers to improve the energy conversion efficiency and found that the addition of Ge, Ti, and Zn can decrease the reflection and slightly increase the flyer velocity. A single coat of black paint has also been applied as the absorption layer of the flyer, but the velocity was not obviously improved. Since these low reflective materials serve not only as an absorption but also an ablation layer, while the interaction material efficiency depends on both the optical and the thermodynamic properties of the flyer material [10], the increase of flyer velocity is limited.

Recently, the use of plasmonic nanomaterials to improve the light absorption through an excitation of localized surface plasmon resonance (LSPR) has attracted considerable interest in the fields of spectroscopic sensors and solar energy conversion [11–13]. Aluminum nanostructures can be used as light-harvesting systems because it covers up a wide spectrum range from ultraviolet to visible light of LSPR [14–17]. Zhang et al. [18] found that an enhancement of 40% in absorption could be achieved by integrating the aluminum particles by using optical simulations. Lee et al. [19] reported a design strategy to achieve a robust platform for plasmon-enhanced light harvesting using aluminum core-shell nanostructures, which resulted in a remarkable increase in photo-to-chemical conversion. Fan et al. [20] demonstrated an ultrafast laser-processing strategy for fabricating highly effective antireflection micro-nano-structures on thick metal surfaces, and an average reflectance of 4.1%, 2.4%, and 3.2% in the broadband spectrum from ultraviolet to near-infrared on Cu, Ti, and W, respectively, surfaces were achieved. However, to our best knowledge, there is no research on using nanostructured material to improve the laser absorption in LDFs.

In this work, we propose a nanostructured aluminum absorption layer in situ prepared on the surface of thin Al flyers to improve the laser absorption and energy conversion efficiency. A femtosecond laser writing technology named as direct laser writing was utilized to fabricated the nanostructures due to its precision, relative simplicity, and high yielding rate [21–23]. The morphology and composition of the surface of the in situ-prepared nanostructures were characterized and their light absorption was tested. To evaluate the energy conversion efficiency of the flyers with a nanostructured absorption layer, the flyers were launched using single-pulsed lasers and their velocities were obtained by a photonic Doppler velocimetry (PDV). Furthermore, the kinetic energy and energy conversion efficiency of the flyers were calculated and discussed.

## Experimental Methods

### Sample Preparation

**Fig. 1:**
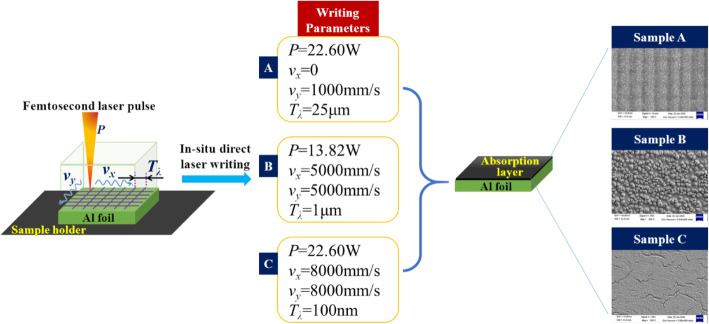
Schematics of the sample preparation method

Al foils with a size of 60 mm × 60 mm × 50 μm (width, length, and height) were used as the reference flyer. These foils were first electro-chemically polished to achieve a low surface average roughness. The nanostructured absorption layers were then in situ prepared on the surface of Al foils by a direct writing laser under an air atmosphere. The direct laser writing used a polarized femtosecond laser (FX200-3-GFH, EdgeWave, Germany) with a wavelength of 1030 nm, pulse duration of 600 fs, and repetition rate of 200 kHz. The output laser power varied from 0 to 100 W. Figure 1 illustrates the direct laser writing preparation process to fabricate the samples. The nanostructures on the surface of Al foils were controlled by changing the radiation laser power and scanning speed and period. Three samples with different nanostructured absorption layers (samples A, B, and C) were prepared. Sample A was irradiated by 22.60-W laser pulses with 1000-mm/s scanning speed in the *y* direction and 25-μm scanning period. Sample B was irradiated by 13.82-W laser pulses with 5000-mm/s scanning speed in both *x* and *y* directions and 1-μm scanning period. Sample C was irradiated by 22.60-W laser pulses with 8000-mm/s scanning speed in both *x* and *y* directions and 100-nm scanning period.

### Characterization Methods

The morphology of the surface of the samples was characterized by scanning electron microscopy (SEM, Ultra 55, Zeiss, Germany) combined with energy dispersive X-ray analysis (EDX, Oxford, Britain). The optical wavelength-dependent reflectivity measurement in the wavelength from 500 to 1500 nm for the samples was carried out with an UV-VIS-NIR spectrophotometer (SolidSpec-3700, Shimadzu, Japan) incorporated with an integrating sphere.

**Fig. 2:**
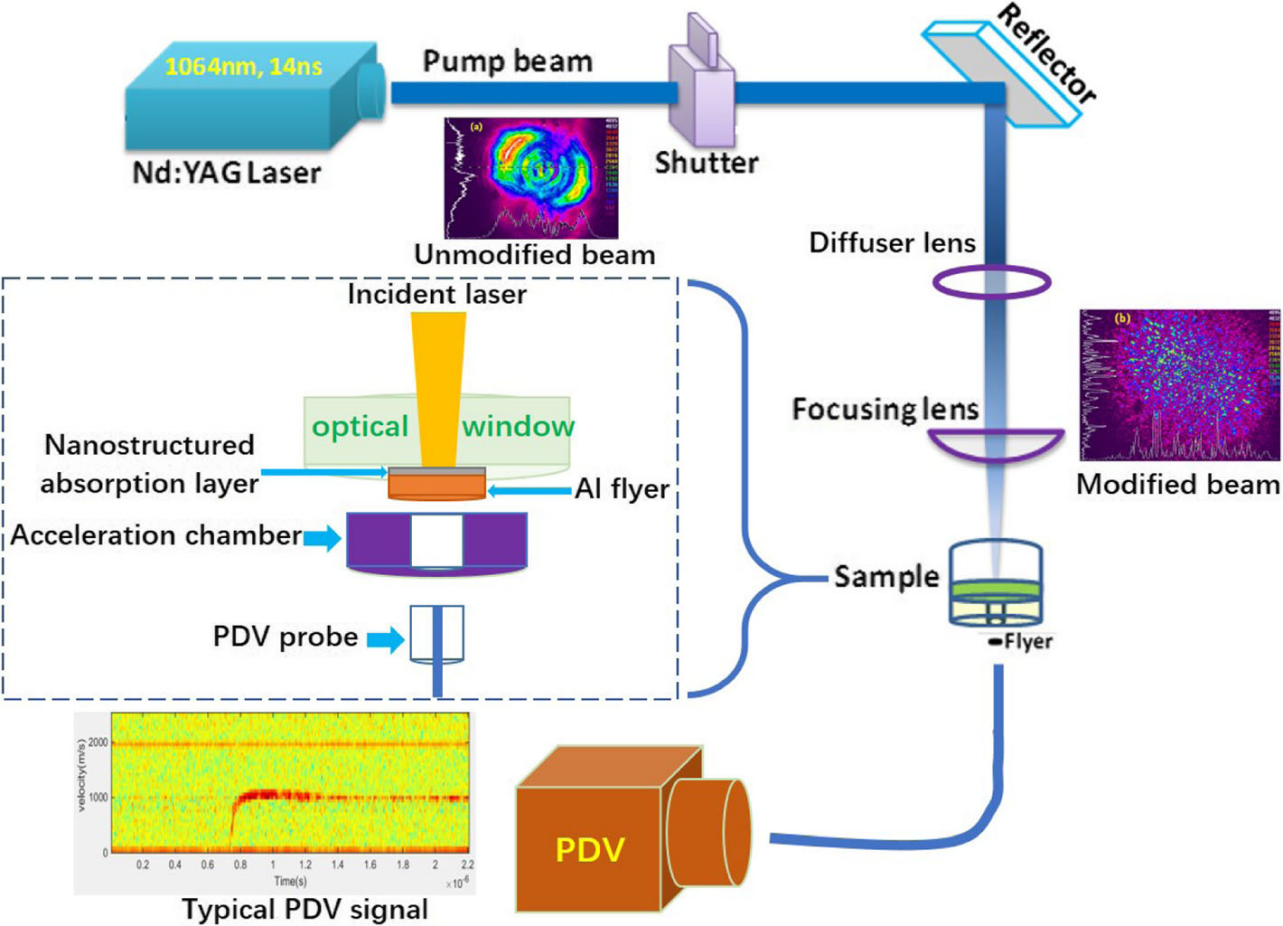
Schematics of the flyer launching system and flyer velocity recording system (PDV)

Figure 2 illustrates the experimental setups used to launch the flyer and characterize the flyer velocity as the velocity is one of the key factors to estimate the flyer performance. A Q-switched Nd:YAG laser (Innolas SpitLight 400, 1064-nm wavelength, 14-ns pulse length) was employed to ablate and launch the prepared samples, and a PDV system is applied to measure the flyer velocity of the samples. The spatial energy distribution of the laser beam was homogenized by a diffusive optic, since the focused beam itself was highly non-uniform. The laser spot had a diameter of 0.5 mm. In the velocimetry experiment, samples were cut into small pieces and adhered onto a sapphire window with the nanostructured layer clung to the window. Steel acceleration chambers with a thickness of 0.2 mm and an inner diameter of 0.6 mm were used. Sixty-millijoule single laser pulses were shot on the samples to produce fast-flying flyers in the acceleration chamber. An optical fiber connected with the PDV system was placed at the exit of the acceleration chamber to record the velocity of the flyer.

## Results and Discussion

### Microstructure of the Absorption Layer

**Fig. 3:**
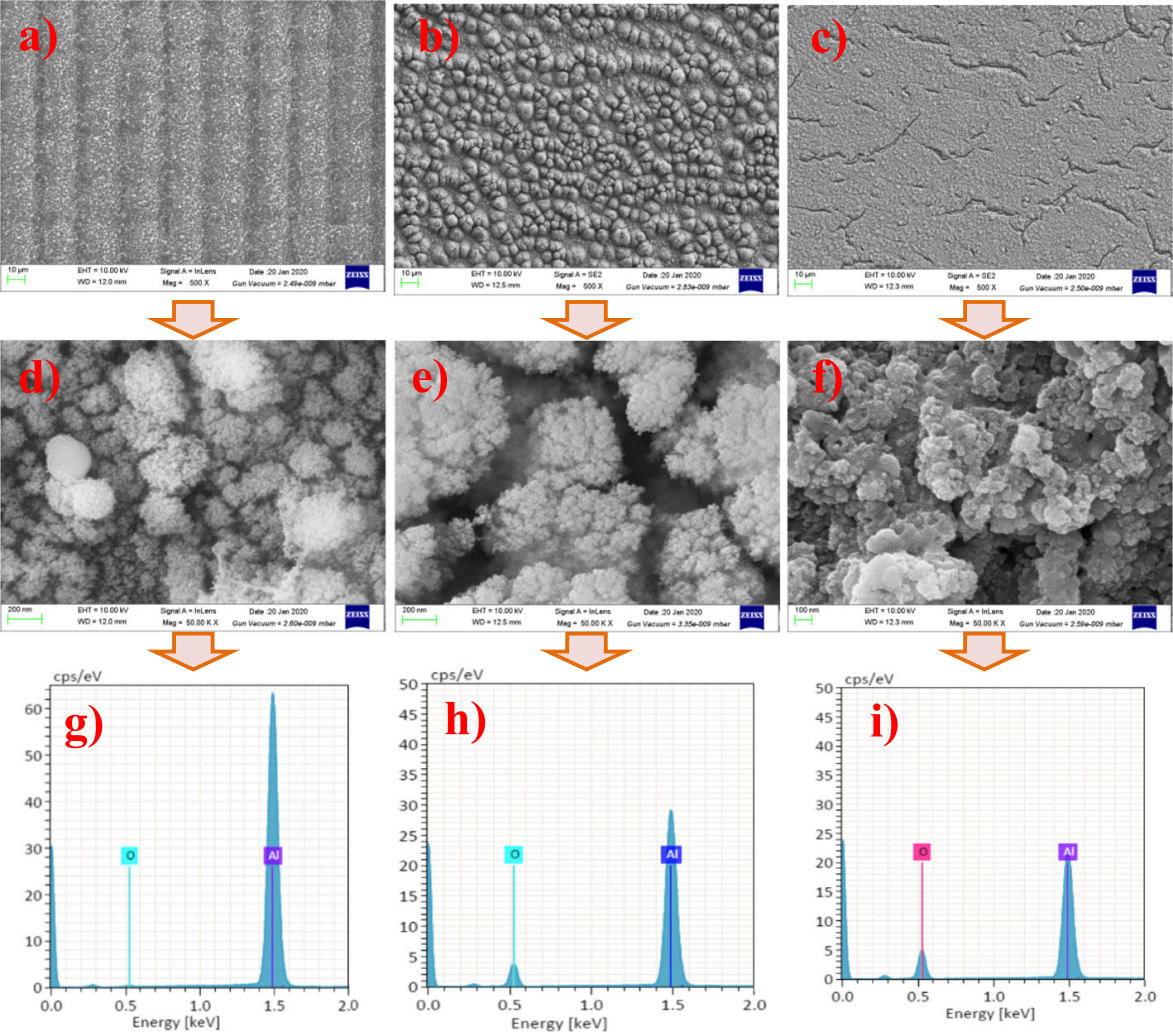
**a** SEM images with × 1000 magnified for sample A. **b** SEM images with × 1000 magnified for sample B. **c** SEM images with × 1000 magnified for sample C. **d** SEM images with × 4000 magnified for sample A. **e** SEM images with × 4000 magnified for sample B. **f** SEM images with × 4000 magnified for sample C. **g** EDX for sample A. **h** EDX for sample B. **i** EDX for sample C

Figure 3a–f show the microstructure of the nanostructured absorption layer of samples A, B, and C. Since sample A was irradiated by ultrafast lasers in one direction with a scanning speed of *v*_*x*_ = 0 and *v*_*y*_ = 1000 mm/s, the surface of sample A exhibits semi-periodic structures, as shown in Fig. 3a. A nano-spherical structure was observed for sample A in Fig. 3d. The nano-spheres with about 50–200 nm diameter were covered with smaller nano-spheres whose diameters were less than 10 nm. Samples B and C were irradiated in both directions and their scanning speeds are much higher than sample A; no evident periodic structures were observed on their surfaces, as shown in Fig. 3b and c. As for sample B, many particles in the scale of micrometers were observed on its surface (Fig. 3b), and the particles were composed of cauliflower nanostructures (Fig. 3e). Since sample C was irradiated and scanned by an even higher speed compared with samples A and B, the accumulation of nanoparticles was much faster and the heat effect was more prominent. Consequently, much thicker nanosheet and nanoparticle aggregations were observed in Fig. 3c and f. And multiple cracks occurred on the surface because relatively high stress emerged during the cooling process due to prominent heat input.

Figure 3g–i are the energy dispersive X-ray analysis (EDX) results for samples A, B, and C, respectively. The EDX showed the presence of Al_2_O_3_ oxides in the composition of nanostructures. The oxides were formed due to the oxidation of aluminum during the laser writing process. The oxygen contents of samples A, B, and C were 2.2, 8.4, and 22.9 atom%, respectively. Apparently, samples B and C had much higher oxygen content compared to sample A, while the irradiation laser power for sample B (13.82 W) was lower than that for sample A (22.60 W) and the irradiation laser power for samples A and C was identical, indicating that the scanning speed and scanning period significantly influence the heat generation and dissipation during direct laser writing. And the oxidation increases with the increase of scanning speed and decrease of the scanning period.

### Light Absorption of the Samples

**Fig. 4:**
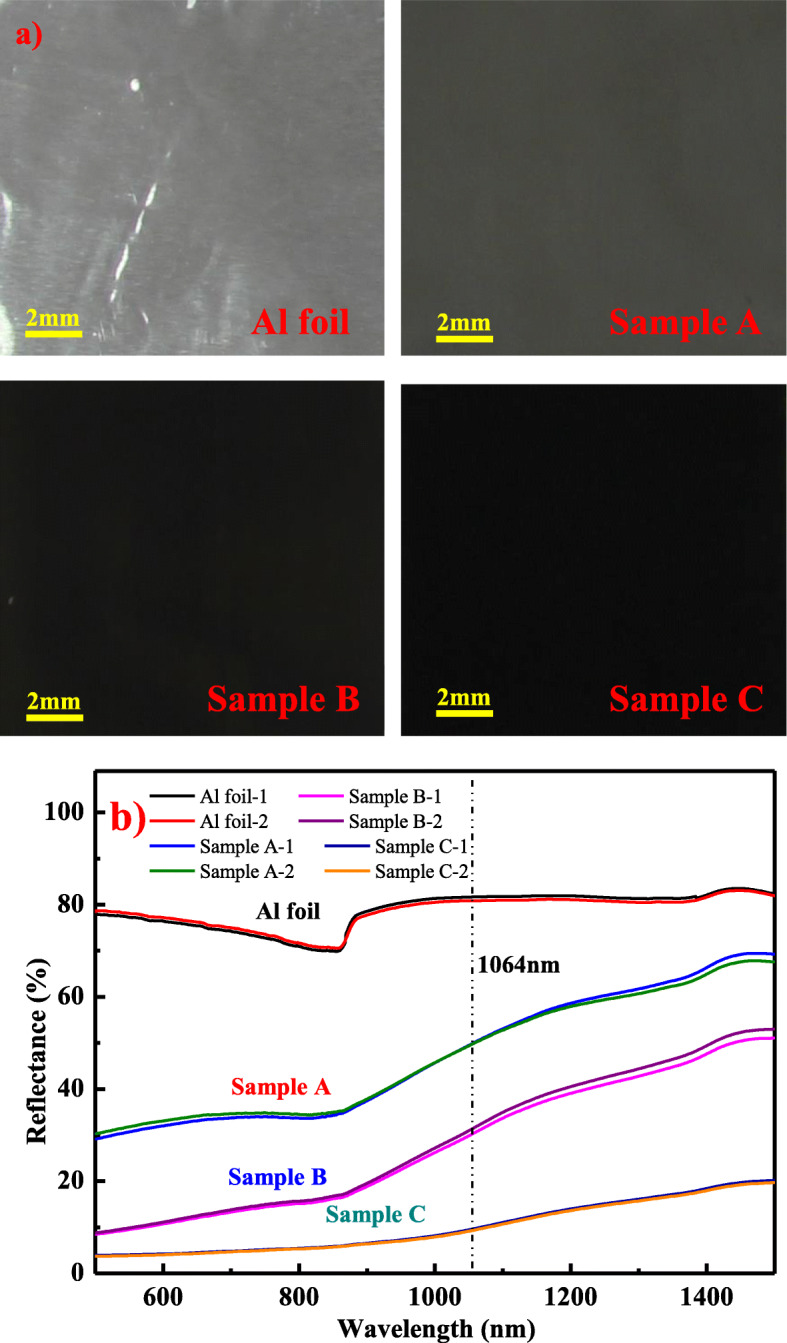
**a** Optical microscope appearances of Al foil and samples A, B, and C. **b** The reflectance spectrum of Al foil and samples A, B, and C

Figure 4a shows the optical microscope appearances of Al foil and the flyers with a nanostructured absorption layer. The color of Al foil is silvery white. With the addition of a nanostructured absorption layer, samples A, B, and C exhibit gray, black, and dark black colors, indicating that more light can be absorbed with the absorption layer. The reflectance of Al foil and samples A, B, and C is tested by a spectrophotometer, and the measurements are repeated two times for each sample. Figure 4b shows the reflectance spectrum of Al foil and the aluminum flyer with nanostructure absorption layer. Since the transmitting thickness of infrared light through metals often varies from a few tens of nanometers to several hundred nanometers [24], thus, none of the light was transmitted through the Al foil samples whose thickness was 50 μm. And the scattered light was included in the reflected light in the measurement using an integral sphere. Consequently, the absorption could be calculated by 1-R (reflectance). Differences were evident between Al foil and the aluminum flyer with a nanostructured absorption layer. The reflectance of Al foil was 81.3% at the laser wavelength of 1064 nm, indicating that 81.3% of incidence light was reflected. The average reflectance can be reduced to 50.5%, 31.5%, and 9.8% for samples A, B, and C, respectively. Therefore, light absorption can be effectively enhanced with the nanostructure absorption layer prepared by direct laser writing. Sample C has the strongest absorption (90.2%) at 1064 nm compared to samples A and B. Aside from the effect of the nanostructures, we believe that the aluminum oxide presented in the nanostructures also tremendously influences the light absorption of the flyer. Generally, Al_2_O_3_ is transparent and does not absorb light; however, in a direct laser writing process, it is highly possible for the generated Al_2_O_3_ and aluminum particles to form a metal-dielectric-metal structure. The structure behaves as an F-P cavity which will in turn enhance the surface plasmon resonance and increase the light absorption [25]. As the oxygen concentrations of samples A and B are far less than that of sample C, implicating that the Al_2_O_3_ particles are richer in sample C than other samples, resultantly, a more enhanced surface plasmon resonance effect and far stronger absorption can be achieved.

### Velocity of the Flyer

**Fig. 5:**
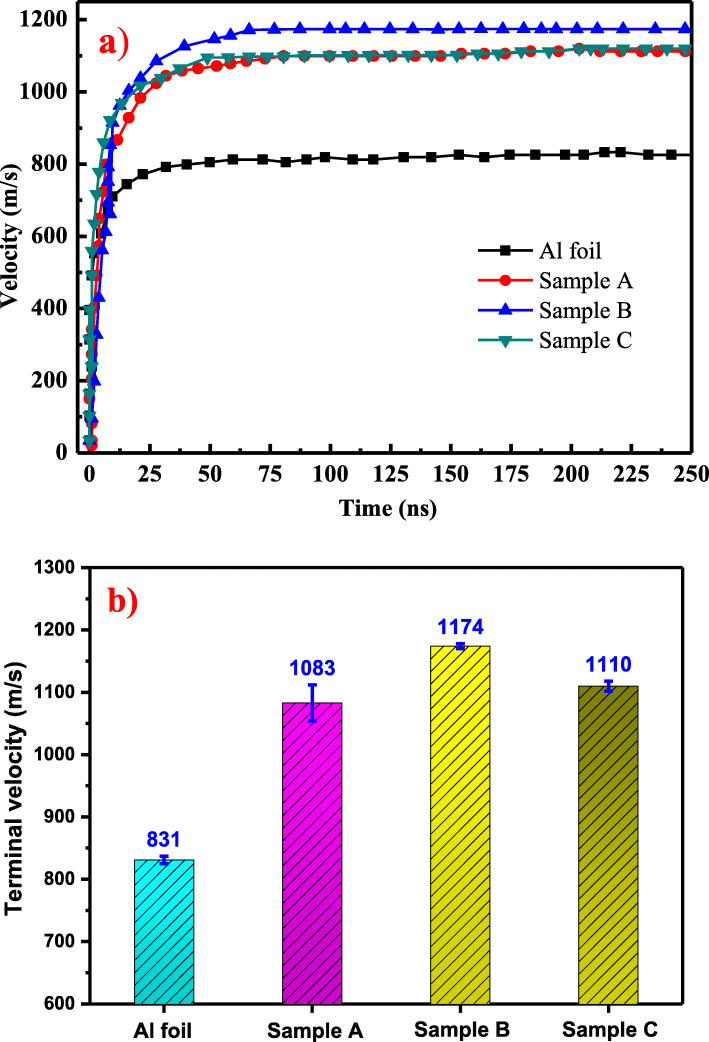
**a** The flyer velocities of Al foil and samples A, B, and C in the acceleration chamber obtained using PDV. **b** The terminal flyer velocities of Al foil and samples A, B, and C

Figure 5 shows the flyer velocities of Al foil and samples A, B, and C. At the beginning of 30 ns, the flyer velocity increases sharply. Afterward, the flyer velocity gradually increases starting from 30 to 200 ns, and hardly changes when the time exceeds 200 ns. The terminal flyer velocity for samples A, B, and C is 1083 m/s, 1173 m/s, and 1110 m/s, respectively, which is about 1.30, 1.41, and 1.33 times higher than that of the Al foil (831 m/s). These results confirmed that the addition of an in situ nanostructured layer can not only enhance the light absorption but also promote the flyer velocity. It is worth mentioning that the flyer velocity for sample B is higher than sample C while sample C has the strongest light absorption. The reason is that sample C has a far richer Al_2_O_3_ content compared with sample B. Ionic bond and metal bond was formed in Al_2_O_3_ and Al, respectively. And it was known that ionic bond was far stronger than metal bond, which makes the vaporization point and melting point for Al_2_O_3_ higher than Al. The melting point and vaporization point for Al_2_O_3_ are 2054 °C and 2980 °C, while the melting point and vaporization point for Al are 660 °C and 2519 °C, respectively. Additionally, the thermal conductivity is 29.3 W/m K and 237 W/m K for Al_2_O_3_ and Al. Hence, it is more difficult for Al_2_O_3_ to vaporize and form plasma at the incident pulsed laser due to its high melting point and low thermal conductivity compared to pure aluminum [26]. Therefore, although the light absorption is enhanced by Al_2_O_3_ in sample C, in the meantime, Al_2_O_3_ consumes some of the incident laser energy while it does not help in driving the flyer.

The kinetic energy of the flyers can be obtained by the following relationship:

1 $$ E=\frac{\left({m}_f-{m}_a\right){v}^2}{2} $$

where *m*_*f*_ is the original flyer mass, and *m*_a_ represents the ablated flyer mass. Moreover, we assume the flyer keeps an integrated state during the flying process. The ablated flyer mass can be evaluated according to the Lawrence and Trott model [27].

2 $$ {m}_a=\frac{\pi {r}^2}{\mu_{\mathrm{eff}}}\ln \frac{\mu_{\mathrm{eff}}{I}_0\left(1-k\right)}{\varepsilon_d} $$

where *r* is the radius of the flyer, *μ*_eff_ is the effective absorption index, *I*_0_ is the incident laser intensity, *k* is the energy loss index, and *ε*_*d*_ is the vaporization energy.

The energy conversion efficiency of the flyer can be denoted using the following equation:

3 $$ \xi =\frac{E_{\mathrm{f}}}{E_{\mathrm{l}}} $$

where *ξ* denotes the energy conversion efficiency of the flyer, *E*_f_ represents the kinetic energy of the flyer, and *E*_l_ represents the incident laser energy.

**Fig. 6:**
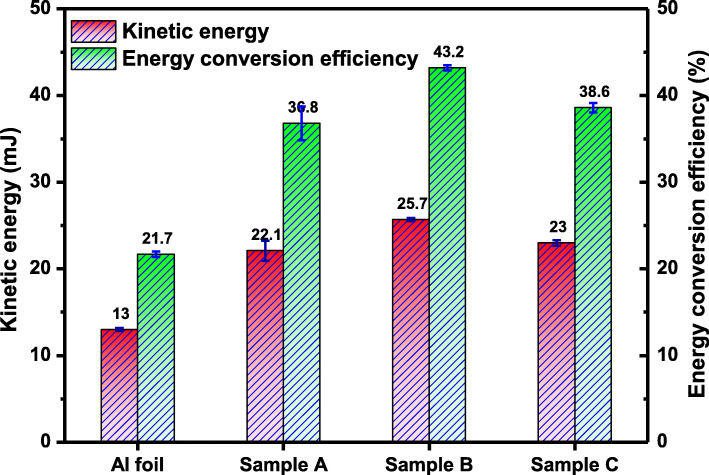
The calculated kinetic energy and energy conversion efficiency of Al foil and samples A, B, and C

The calculated results of the flyer kinetic energy and energy conversion efficiency were illustrated in Fig. 6. The energy conversion efficiency for samples A, B, and C is 36.8%, 43.2%, and 38.6%, respectively, which is 1.70, 1.99, and 1.78 times that of the Al foil (21.7%). In this work, when a nanostructured absorption layer is added on Al foil, the highest energy conversion efficiency almost doubled. The experimental results are summarized in Table 1. Therefore, the in situ fabrication of a nanostructured absorption layer on the surface of a flyer provides a new method to significantly improve the energy conversion efficiency of a LDF.

**Table 1:** Summarization of the experimental results

Sample	Laser energy (mJ)	Reflectance (%)	Velocity (m/s)	Kinetic energy (mJ)	Conversion efficiency (%)
Al foil	60	81.3	831	13.0	21.7
Sample A		50.5	1083	22.1	36.8
Sample B		31.5	1173	25.7	43.2
Sample C		9.8	1110	23.0	38.6

## Conclusions

Nanostructured absorption layers were successfully in situ prepared on the surface of thin Al foils by direct laser writing technology. Furthermore, we demonstrated that through controlling the laser pulse injection, both the microscale and nanoscale structural features can be realized. Consequently, a substantial decrease in light reflectivity and a significant enhancement in light absorption can be realized. By in situ preparing the nano-absorption layer on the surface of an Al foil, the light absorption can be increased from 18.7 to 90.2%. The increase in light absorption will in turn result in an evident increase in the velocity and kinetic energy of a laser-driven flyer. The energy conversion of the flyer with nanostructured absorption layer can be significantly improved compared with Al foil, the max energy conversion in this study reaches up to 43.2% which is 1.99 times that of the Al foil (21.7%). Therefore, the aluminum nanostructure absorption layer in situ prepared on the surface of the flyer provides a new method to increase the absorption of laser energy and improve the energy conversion efficiency of a LDF. Moreover, the in situ preparation technology present in this work is also promising in fields of photochemistry, sensing, photodetectors, and quantum optics.

## Supplementary information

**Additional file 1..**

## Data Availability

All authors declare that the materials, data, and associated protocols are available to the readers, and all the data used for the analysis are included in this article.
