# Supplementary material for: Improving the Energy Conversion Efficiency of a Laser-Driven Flyer by an In Situ-Fabricated Nano-absorption Layer
Source: Nanoscale Res Lett. 2020 Jun 5;15:125. doi: 10.1186/s11671-020-03346-5 (PMC7275114; doi:10.1186/s11671-020-03346-5)
Supplement: Supplementary file 1 — Additional file 1.. [file 11671_2020_3346_MOESM1_ESM.docx]

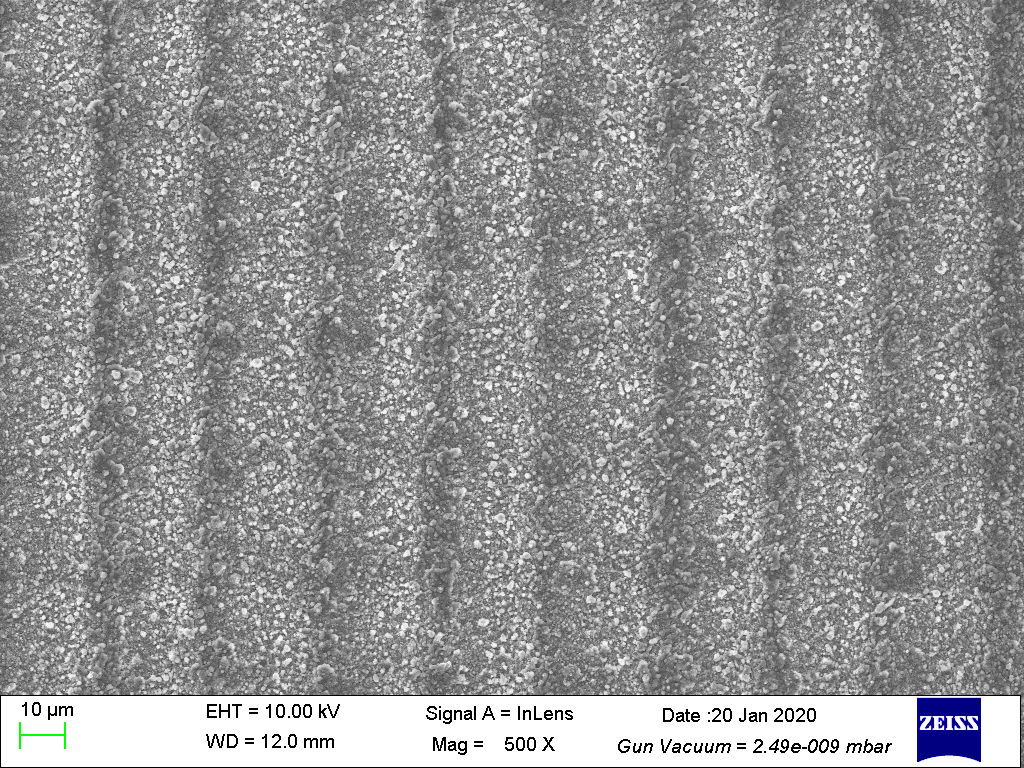


**a)**

**Fig. 3a** SEM images with 1000X magnified for Sample A.


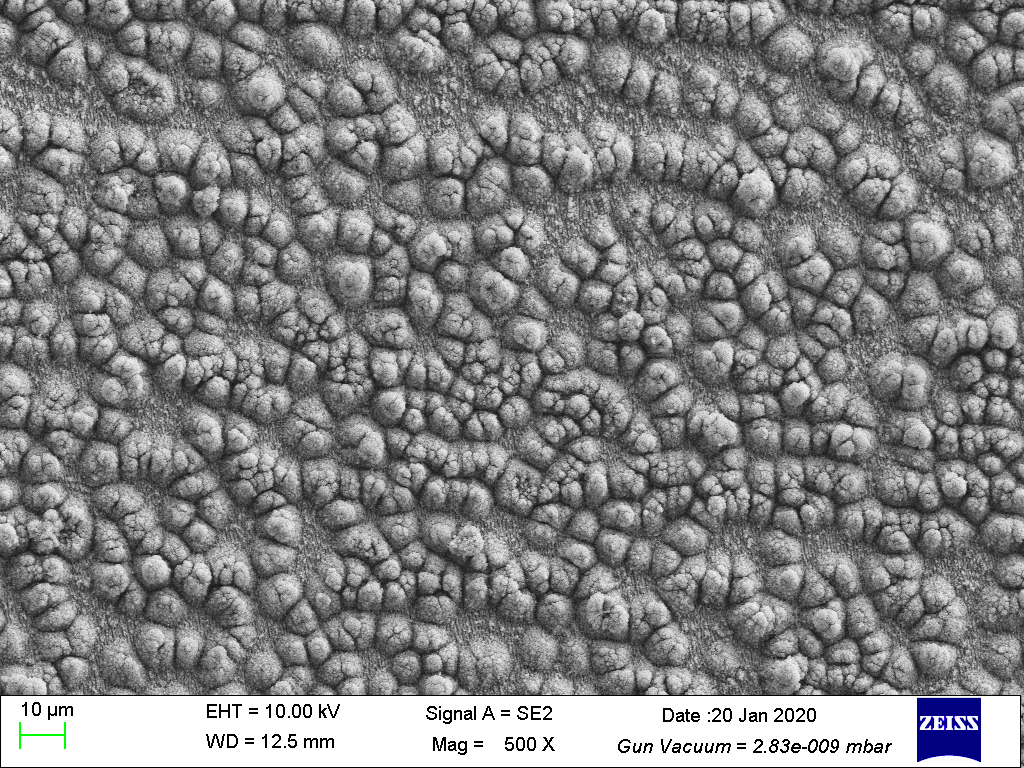


**b)**

**Fig. 3b** SEM images with 1000X magnified for Sample B.


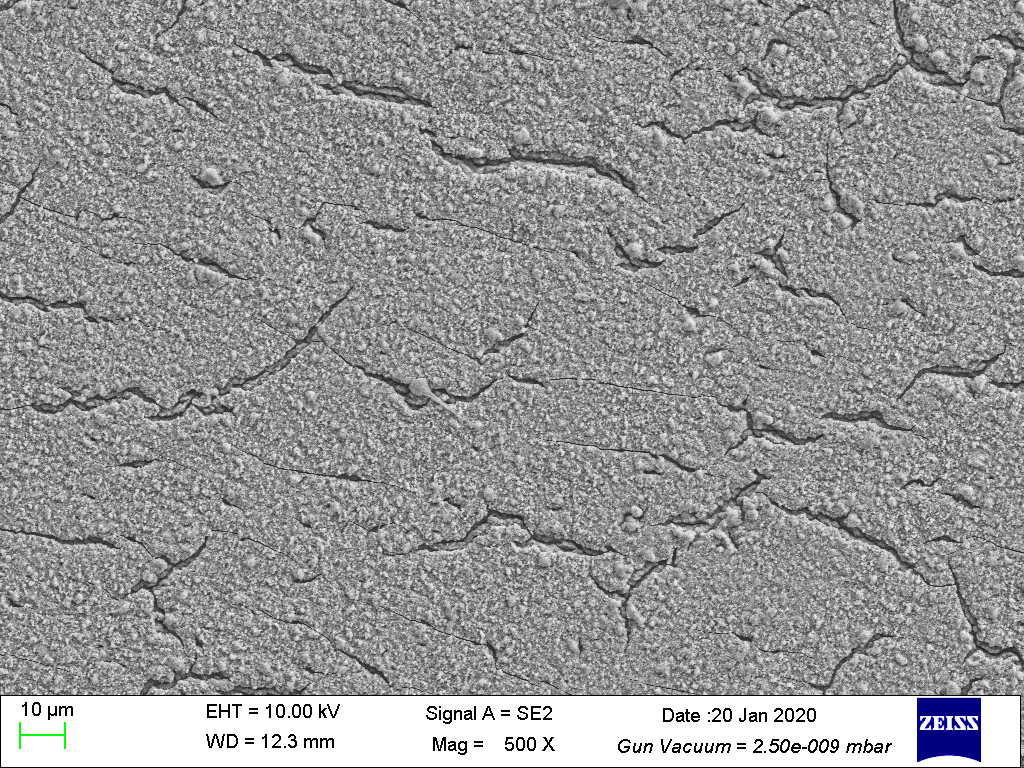


**c)**

**Fig. 3c** SEM images with 1000X magnified for Sample C.


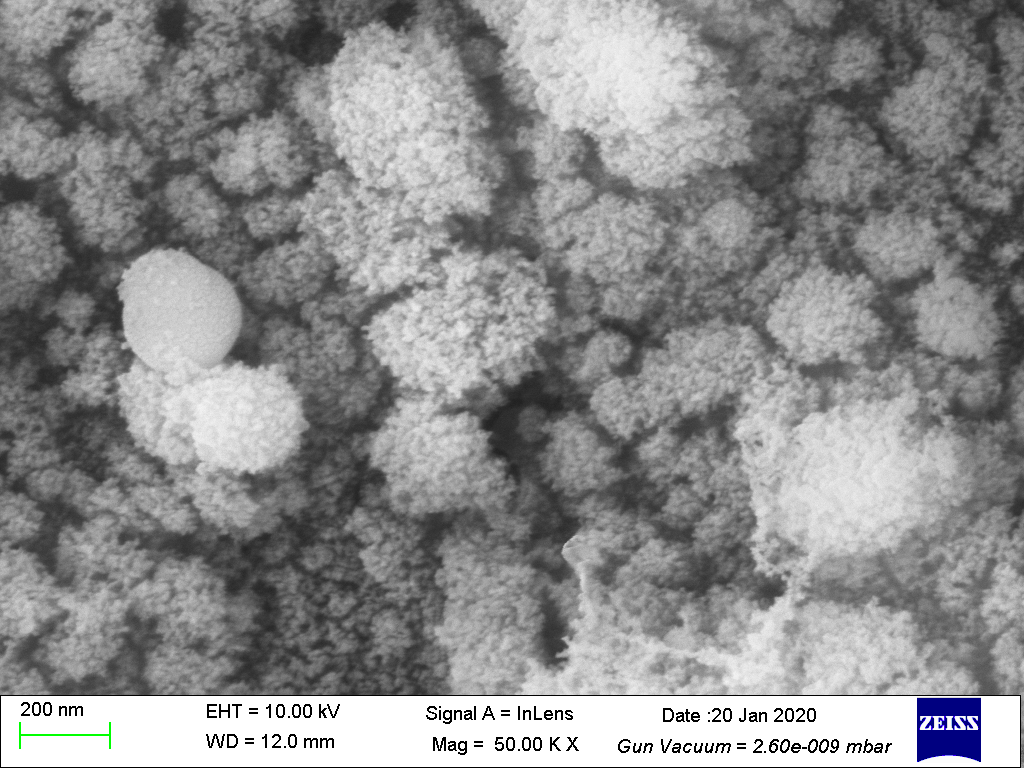


**d)**

**Fig. 3d** SEM images with 4000X magnified for Sample A.


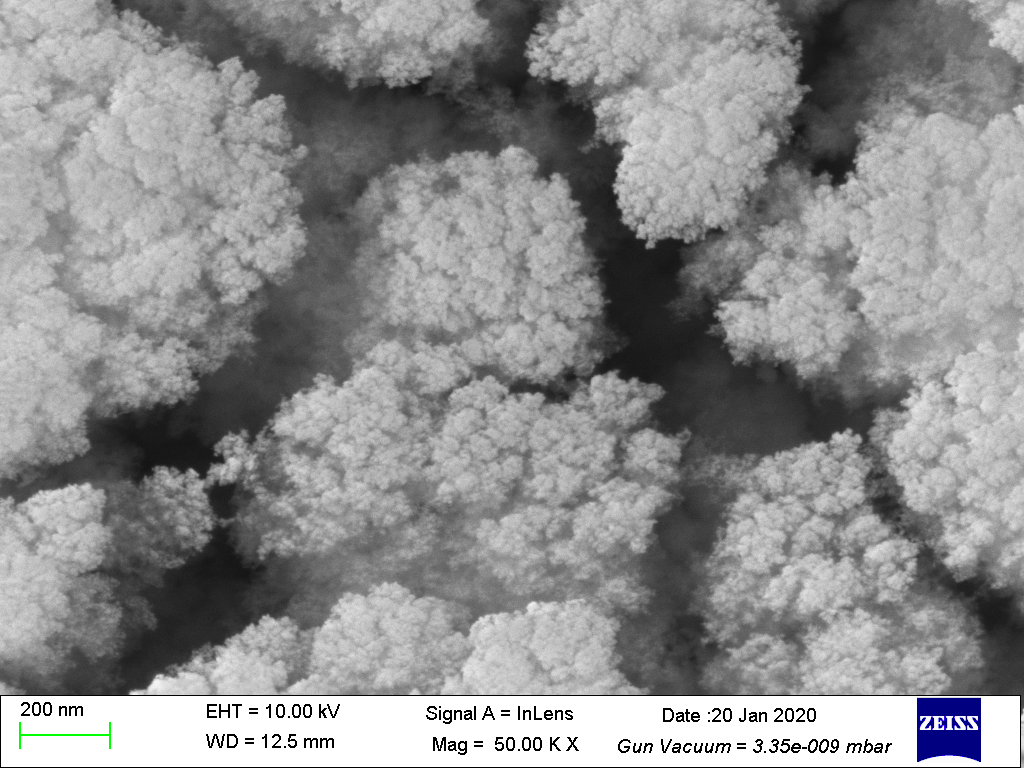


**e)**

**Fig. 3e** SEM images with 4000X magnified for Sample B.


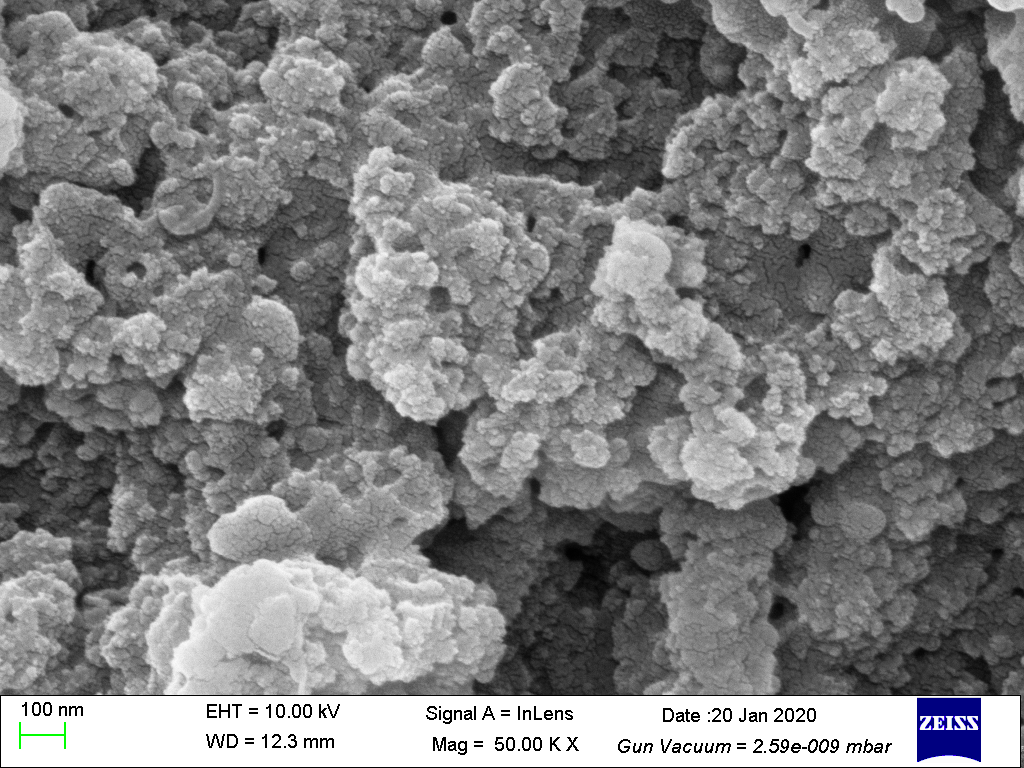


**f)**

**Fig. 3f** SEM images with 4000X magnified for Sample C.

**Fig. 5b** The flyer velocities of Al foil and Sample A, B and C in the acceleration chamber obtained using PDV (repetitively).
